# Modelling accidental hypothermia effects on a human body under different pathophysiological conditions

**DOI:** 10.1007/s11517-017-1657-3

**Published:** 2017-06-05

**Authors:** Alberto Coccarelli, Etienne Boileau, Dimitris Parthimos, Perumal Nithiarasu

**Affiliations:** 1 Division of Cancer and Genetics, School of Medicine, Cardiff University, Cardiff, CF14 4XN UK; 2 Biomedical Engineering and Rheology Group, Zienkiewicz Centre for Computational Engineering, Swansea University, Swansea, SA2 8PP UK

**Keywords:** Hypothermia modelling, Bio-heat transfer, Thermoregulation, Cardiomyopathy, Malnutrition

## Abstract

Accidental exposure to cold water environment is one of the most challenging situations in which hypothermia occurs. In the present work, we aim to characterise the energy balance of a human body subjected to such extreme environmental conditions. This study is carried out using a recently developed computational model and by setting boundary conditions needed to simulate the effect of cold surrounding environment. A major finding is the capacity of the body core regions to maintain their temperature high for a substantial amount of time, even under the most extreme environmental conditions. We also considered two disease states that highlight the spectrum of possible pathologies implicated in thermal regulation of the human body. These states are (i) cardiomyopathy, which affects the operating capacity of the heart, and (ii) malnutrition, which directly impairs the body’s ability to regulate heat exchange with the environment. We have found that cardiomyopathy has little influence on the thermal balance of the human body, whereas malnutrition has a profound negative effect on the thermal balance and leads to dramatic reduction in core temperature.

**Electronic supplementary material:**

The online version of this article (doi:10.1007/s11517-017-1657-3) contains supplementary material, which is available to authorized users.

## Introduction

Although rare, accidental hypothermia can lead to significant morbidity and mortality [1]. The human body is considered to have developed hypothermia if the core body temperature reaches a value below 35 ^∘^C [2]. The hypothermia may be categorised into primary and secondary types. The primary type occurs when a human body’s heat balancing mechanisms are working properly but are subjected to extreme cold conditions, typically involving exposure to cold air or immersion in cold water. The cold air usually takes several hours to cause hypothermia but immersion hypothermia develops rapidly. This is mainly due to the higher convective heat transfer coefficient of water. Secondary hypothermia affects people whose heat balancing mechanisms are impaired in some way or another and cannot respond adequately to moderate or perhaps even mild cold weather. This condition often affects patients suffering from stroke, diabetes, malnutrition, bacterial infection, thyroid disease and spinal cord injuries. Secondary hypothermia may also pose a threat to the elderly, who are on medications or suffering from illnesses that affect their ability to conserve heat. Other influencing factors that can put the elderly at risk are malnutrition and immobility.

The hypothermia can also be categorised based on core body temperature as mild (33–35 ^∘^C), moderate (28–32 ^∘^C), severe (24–28 ^∘^C) and no vital signs (< 24 ^∘^C) [2, 3]. The impact of hypothermia on the nervous system often becomes apparent quite early. Coordination, for instance, may begin to suffer as soon as the body temperature reaches 35 ^∘^C (95 ^∘^F). The early signs of hypothermia also include cold and pale skin and intense shivering, which stops between 32.2 ^∘^C (90 ^∘^F) and 30 ^∘^C (86 ^∘^F). Other harmful consequences include muscle rigidity, dehydration as well as liver and kidney failure. Frank et al. [4] investigated the hemodynamic response to induced mild hypothermia conditions. This study has shown that when core temperature reaches a threshold (∼ 1^∘^C below the normothermic baseline), pressure amplitude (PA) and heart rate (HR) rise up (by + 23 *%* and + 16 *%*, respectively). It was also noticed that the respiratory rate rises. When subjected to severe hypothermia, the thermoregulatory system decreases the blood flow towards the periphery of the body in order to avoid damage to the vital inner organs. The heart and respiratory rates and blood pressure fall once the 32.2 ^∘^C (90 ^∘^F) mark is passed [3]. Below 30 ^∘^C (86 ^∘^F) most victims reach a comatose state, and below 27.8 ^∘^C (82 ^∘^F) the heart’s rhythm becomes dangerously disordered. The findings in [5] indicate that the sympathetic nervous system responds quickly to hypothermia but may be “switched off” at a threshold temperature of about 29 ^∘^C.

As discussed, the mechanism of hypothermia is extremely complex and involves the interaction of the nervous system and various processes of the human body. In the recent past, some relevant theoretical studies concerning induced hypothermia have been carried out [6–8]. However, they are restricted to the head region. We remark that it is extremely difficult to precisely represent each process and interaction mathematically. It is also computationally very expensive to model a fully three-dimensional body to understand the energy transport.

To overcome these difficulties, we employ a framework [9] that accounts for blood flow and heat transfer in tissues based on a reduced-order model. Such a tool is a good compromise between accuracy and cost to study accidental hypothermia. In the proposed model, the systemic blood circulation in the arterial tree is modelled using one-dimensional Navier-Stokes equations and the Taylor Galerkin finite element method is used to solve the equations [10]. To study the energy transport throughout the body, the arterial tree is coupled with a multi-segmental bioheat model, representing solid tissues, of the human body.

In the current work, we modify the abovementioned computational model by setting specific inner and boundary conditions in order to reproduce the body’s cold water immersion. With this bioheat transfer model, we characterise the thermal transient occurring in a human body during the development of hypothermia. To the authors’ knowledge, no studies based on a human computational model have been proposed so far for studying hypothermia. At first, we carry out a sensitivity analysis on the time taken by a human body to reach mild hypothermic condition. We also analyse the temporal evolutions of blood and tissue temperatures.

In addition to studying a normal human body, the present work also investigates the effects of specific abnormalities on reaching hypothermic conditions. A detailed temperature map presented here clearly brings out the differences between normal and abnormal conditions. In addition to deriving such differences, a better understanding on the body’s response may also be extremely helpful in therapeutic hypothermia, which represents an effective treatment for a wide array of clinical problems [11, 12].

## Methods

The model consists of three main components: a network of elastic tubes representing blood flow in large arteries, 14 solid cylinders for accounting for the tissue and a thermoregulatory system. In the following subsections, we briefly present each subsystem and how they are linked. For further details, readers are referred to [9, 10, 13–15] and the supplementary material provided.

### Arterial blood flow

To represent the larger arterial system, the model proposed by Low et al. [13] is adopted. This is a branching network characterised by bifurcations and cross-sectional discontinuities. The reflections due to network singularities and terminals are also incorporated into the model. The fluid system is described by three variables: the cross-sectional area (*A*), the average values of velocity (*u*) and temperature (*T*) over the cross section. The flow is considered incompressible, laminar and pressure (*p*) is linked to area via a non-linear relationship proposed by Formaggia et al. [16] and Olufsen et al. [17]. For the flow mass and momentum equations, we followed [10, 18]. Energy conservation for fluid system is established via [14, 19]:

1 $$ \frac{\partial{{T}}}{\partial t}+{{u}}\frac{\partial {\textit{T}}}{\partial x} +\alpha \frac{\partial^{2} {{T}}}{\partial x^{2}}= +\frac{2 h_{con,in}}{\rho c_{p} \sqrt{A/\pi}}(T_{w}-T)  $$

where *T*_*w*_ is the temperature of the first tissue node that is interacting with fluid, *h*_*con*,*in*_ is the heat transfer coefficient between fluid and wall while *α*, *ρ* and *c*_*p*_ are, respectively, the fluid thermal diffusivity, density and specific heat.

In the flow model employed, the density (*ρ*) and viscosity (*μ*) are assumed to be constant. The thermal properties, specific heat (*c*_*p*_) and thermal conductivity (*k*), of the blood are also assumed to be constant. The energy conservation equation can be written with the mass and momentum in the following compact form:

2 $$ \frac{\partial \bar{\mathbf{U}}}{\partial{t}}+\mathbf{H}\frac{\partial \bar{\mathbf{U}}}{\partial x} +\frac{\partial \bar{\mathbf{G}}}{\partial x}= \bar{\mathbf{S}}  $$

with:

3 $$\begin{array}{@{}rcl@{}} \bar{\mathbf{U}}&=&\left[\begin{array}{l} A \\ u \\ T \end{array}\right];~~~ \mathbf{H}=\left[\begin{array}{lll} ~~~~u & A & 0 \\ {\frac{\beta}{2\rho\sqrt{A}}}& u & 0\\ ~~~~0& 0& u\end{array}\right]; \\ \bar{\mathbf{G}}&=&\left[\begin{array}{l} ~~~~~0 \\ ~~~~~0 \\ - \frac{k}{\rho c_{p}} \frac{\partial T}{\partial x}\end{array}\right]~~~\text{and}~~~ \bar{\mathbf{S}}=\left[\begin{array}{l} ~~~~~~~~~~~~~~~~~0 \\ ~~~~~~~~-{8\pi\mu \over \rho} \frac{u}{A} \\ \left( \frac{2 h_{con,in}}{\rho c_{p} \sqrt{A/\pi}}\right)(T_{w}\!-T)\end{array}\right]\\ \end{array} $$

where $\bar {\textbf {U}}$, $\bar {\textbf {G}}$ and $\bar {\textbf {S}}$ are the vectors of primitive variables, the diffusive and source terms, respectively, and **H** is the Jacobian matrix.

The conservation laws for flow in large arteries can be written in terms of characteristic variables. They are useful for prescribing inlet and outlet variables and also for transmitting information in discontinuities between segments [10]. The aortic valve is modelled at the fluid inlet point, while the pumping action of the heart is modelled as a prescribed forward pressure source [10, 13]. To model branch ending, tapering vessels are used. At the inlet, the temperature is set equal to the interacting tissue temperature. At exit nodes, temperature is extrapolated if velocity is positive, otherwise it is set equal to that of the surrounding tissue. The numerical scheme used for solving the set of flow and temperature equations is the explicit form of locally conservative Taylor Galerkin method (LCG) [9, 14, 15, 20, 21].

### Solid tissues

**Fig. 1:**
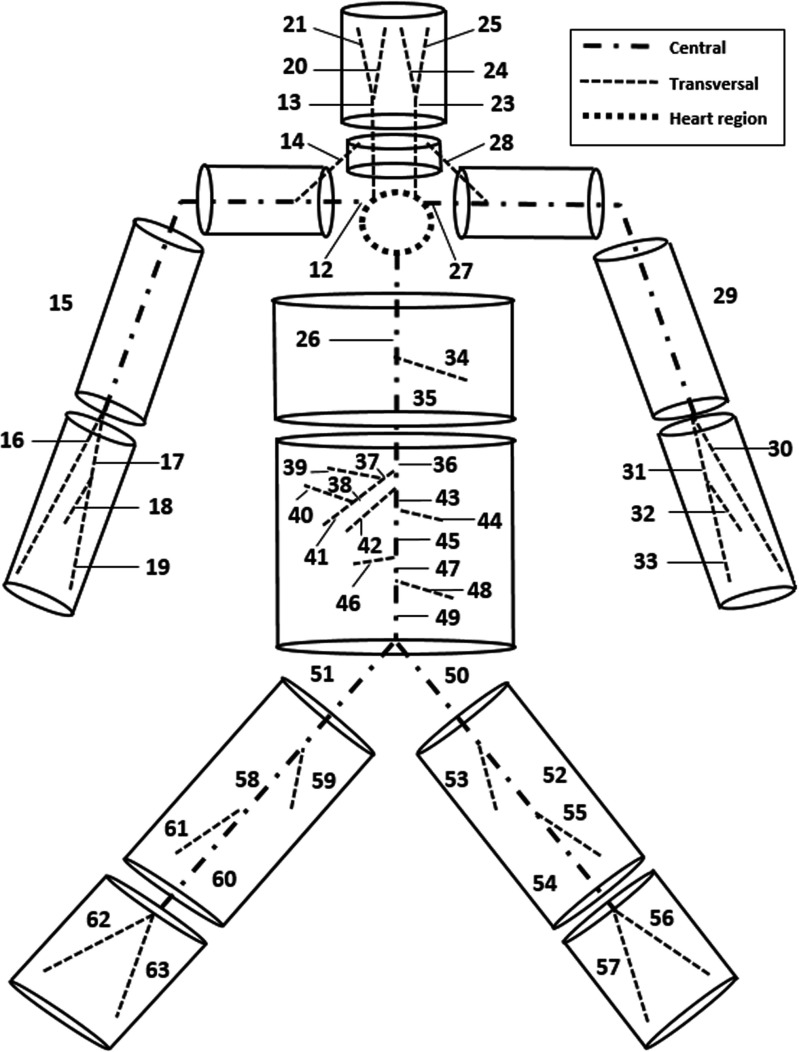
Schematic representation of the passive system, constituted by an arterial network embedded into a body soft tissue. The arterial tree is taken from [13] and consists of 63 segments plus tapering vessels at the extremities. The tissue model is built on the vacular framework [9] by using solid cylinders

The solid tissue representation is carried out by dividing the body into 14 multilayered cylindrical elements representing the head, neck, shoulders, thorax, abdomen, thighs, legs, arms and forearms [22]. The coupling between solid and arterial systems is shown in Fig. 1. The heat transfer between arteries and veins is not considered as blood velocity in the veins is significantly lower than that of arteries and the temperature of the venous flow is close to that of the surrounding tissue. The heart represents the inlet of the fluid network and is not included in any of the cylinders. Large arteries proposed in [10] are subdivided into three categories: heart region, central and transversal. For the heart region, adiabatic and isothermal conditions are assumed. Every central artery is localised along one or more cylinder axes, while each transversal one crosses transversely one or more cylinders. The cylinder discretisation in the radial direction starts from one-dimensional fluid mesh. If we consider the cylinder as a collation of axial slices, the central vessel node of a slice is always the innermost solid node. By comparison a transversal vessel nodes lie on solid nodes that are away from central vessels. The radius allows to localise the tissue nodes in which the transversal vessel lies. In order to evaluate tissue temperature (*T*_*t*_), the one-dimensional bioheat transfer equation in cylindrical coordinates is solved along the radial direction:

4 $$ \rho_{t} c_{t} \frac{\partial T_{t}}{\partial t} - k_{t} \frac{1}{r} \frac{\partial}{\partial r}\left( r \frac{\partial T_{t}}{\partial r}\right)=q_{m} +\phi \rho c_{p}(T-T_{t})  $$

where *q*_*m*_ represents the volumetric heat generation, *ϕ* is the perfusion coefficient while *ρ*_*t*_, *k*_*t*_, *c*_*t*_ are the tissue density, specific heat and conductivity. The thermomechanical properties reported above vary depending on the tissue. The metabolic heat source *q*_*m*_ and diffusion parameter *ϕ* may be affected by the thermoregulatory system while the others (*ρ*_*t*_, *k*_*t*_, *c*_*t*_) are time independent.

We assume that tissues receive/lose heat from large arteries by convection and from small ones by perfusion. For a cylindrical section, convection contribution is evaluated at the nodes in which vessels are localised. For central vessel nodes, prescribing inner convection is straightforward, while for the transversal ones, it is slightly different. We assume that convection contributions of transversal arteries may be incorporated as an inner heat source of the solid node on which they lie. The perfusion is modelled through perfusion coefficients and assumed to be proportional to the temperature difference between average, weighted, larger arterial and the tissue temperatures. Under exercise conditions, an increment in metabolic activity occurs and it is modelled by increasing the volumetric heat generation in the muscle involved.

The conduction problem in the tissue along the radial direction is solved using the forward Euler method. As the domain accounts for more than one tissue layer, continuity of flux is imposed at the interface between two layers. The flux exchanged between the skin layer and the outside environment is the sum of the convection losses to the ambient air, radiation losses with surrounding surfaces and sources and evaporation of moisture from the skin. The respiration losses are incorporated by considering a negative volumetric heat source (*q*_*bre*_) at all lung nodes (for more details see [23]).

### Control system

According to [3], a state of thermoneutrality exists when the core and mean skin temperatures are, respectively, 36.8 ^∘^C and 33.7 ^∘^C. If thermoneutrality is not satisfied, different mechanisms may be triggered to balance heat. We define the core temperature (*T*_*core*_) as the averaged values over all nodes contained in the first layer of the head, neck, thorax and abdomen, while the mean skin temperature (*T*_*skin*_) as the averaged temperature over all outer skin nodes. We point out these variables are global values and not evaluated locally at each node.

Whenever core temperature drops below a determined threshold, shivering occurs. Thus the volumetric heat generation rises linearly until core temperature increases to a lower saturation threshold. After such limit has been reached, all metabolic activities will be reduced. By adopting the methodology proposed in [23], we can evaluate the global body energy production by shivering, which depends on both *T*_*core*_ and *T*_*skin*_. The shivering heat per unit volume (*q*_*shiv*_) may then be obtained by dividing the total segmental heat production by muscle volume. This extra source contribution is added to *q*_*m*_ in Eq. 4. Sweating occurs for an increased skin temperature and involves latent heat losses at the external surface of the body. For modelling such mechanism, we followed what is proposed in [24]. The vasoconstriction and vasodilatation of skin vessels allows the amount of flow to vary and thus heat transport in the peripheral regions. For modelling such mechanisms, we adopted again the methodology proposed in [23]. In this case it is assumed that the perfusion ratio at skin nodes depends directly on the core and skin temperatures. The corresponding perfusion rate *ϕ* is evaluated by dividing the flow rate by the skin mass of the segment considered. For the sake of simplicity, we assume that breathing losses vary linearly (−6.53 *%*/^∘^C) with the core temperature (this rate has been evaluated from [25]).

### Subsystem interconnections

**Fig. 2:**
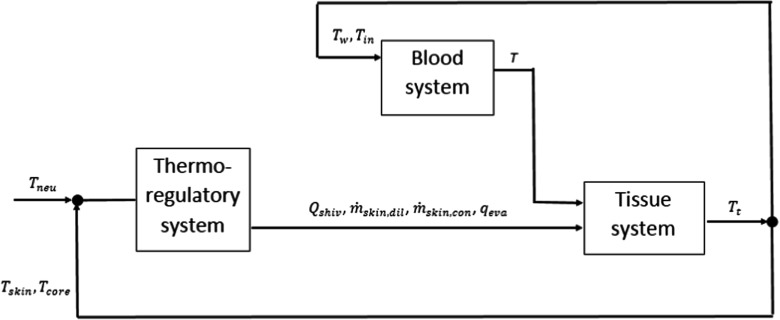
Active and passive systems interconnections. The thermoregulatory system modulates shivering (*Q*_*shiv*_), vasomotion ($\dot {m}_{skin,dil}$, $\dot {m}_{skin,con}$) and sweating (*q*_*eva*_) according to *T*_*core*_ and *T*_*skin*_ variations. These latter quantities are evaluated globally from tissue temperature (*T*_*t*_) field. The blood system is not directly affetcted by the thermoregulatory system but it is coupled with tissue system by means of the wall temperature (*T*_*w*_) and the inlet circuit temperature (*T*_*in*_), both derived from (*T*_*t*_) field. The blood temperature (*T*) computed in the fluid system serves as input in the solid model

Here, we describe how we structured our model. More precisely, we present all existing interconnections between all subsystems (see also Fig. 2). The thermoregulatory response is evaluated by knowing *T*_*core*_ and *T*_*skin*_ of the previous time step and comparing them with thermoneutrality reference values (*T*_*neu*_). Such control system is able to modify tissue balance through shivering heat source, increment or decrement of skin perfused flow and sweating losses. In the present model, it is assumed that such a thermoregulatory system does not affect the inlet pressure signal for the arterial system. As blood variables are evaluated in an explicit way, we can use *T*_*w*_ of the previous time step in the calculations. The fluid inlet temperature prescription is made with *T*_*t*_ (tissue temperature) of the previous time step as well. Once blood system outputs are calculated, it is possible to compute the tissue temperatures before starting a new iteration. The whole system is schematically presented in Fig. 2.

## Results and discussions

### Settings

For simulating immersed hypothermia, we need to specify appropriate settings in the model. In the case studied, sweating and radiative contributions on the thermal balance are assumed to be negligible. The model proposed in [9] is used here and it accounts for a comprehensive theremoregulatory system which includes vasodilation and vasoconstriction mechanisms. With regard to breathing, the inhalated air is assumed to have the same temperature as that of surrounding water. For prescribing a pressure signal in the left ventricle (LV), we adopt the method proposed in [10, 13]. The blood properties are reported in Table 1. As in [14], the boundary conditions for fluid energy conservation are provided by local interacting wall temperatures. The initial tissue and blood temperatures (*T*_*t*_ and *T*, respectively) are set at the quasi-steady state conditions obtained for a body in an environment at 20 ^∘^C and 30% of relative humidity (R.H.). By using an arterial network [13], flow results can be monitored at several monitoring points along the tree. Here, blood temperature is recorded at four points along the arterial tree, respectively, at the middle of abdominal aorta II, left external carotid, right radial and right external iliac arteries (for more details about vessel labelling see [13]). The tissue temperatures are recorded for the sections corresponding to the blood nodes of the abovementioned arteries.

**Table 1:** Fluid parameters and properties used in the simulations

Density of fluid, *ρ*(*g*/*c* *m* ^3^)	1.06
Viscosity of fluid, *μ*(*p* *o* *i* *s* *e*)	3.5 ×10^−2^
Thermal conductivity of fluid, *k* (*W*/*c* *m* ^∘^C)	0.05
Specific heat of fluid, *c*_*p*_ (*J*/*g* ^∘^C)	3.9

### Immersion in treading water of a normal human body

**Fig. 3:**
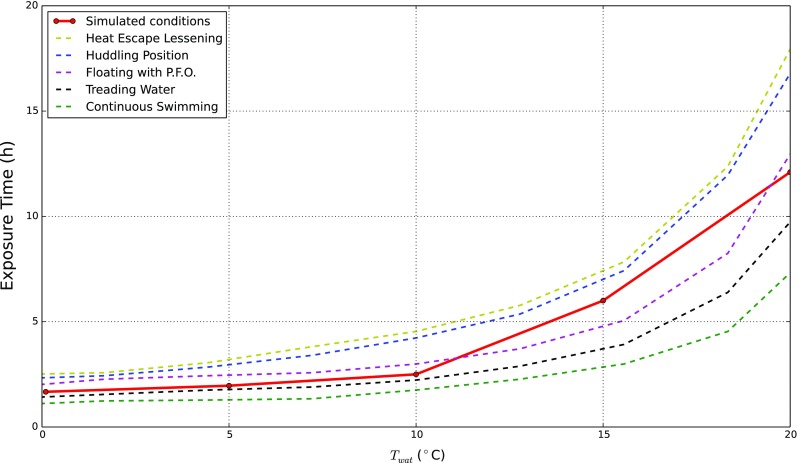
Hypothermia - median lethal exposure. The chart shows the time necessary to develop lethal hypothermia for different swimming water conditions. *Dashed lines* are generated from the experimental measurements given by [26]. The *dark red line* is built on five different simulation results

Simulations were carried out considering five different water temperatures (*T*_*wat*_): 0.1, 5, 10, 15 and 20 ^∘^C. The reference convective heat transfer coefficient between water and skin (*h*_*con*,*out*_) is assumed to be equal to 0.100 *W*/(*K* *c* *m* ^2^) (corresponding to “treading water” condition). For evaluating the time required to reach hypothermic condition, we assume that clinical hypothermia occurs when core temperature goes below 35 ^∘^C (mild hypothermia). From [3, 5] we assume that the exposure becomes “lethal” when the core temperature reaches 28 ^∘^C. As reported in [26], the lethal exposure time is related to water temperature and how long the body is exposed as shown in Fig. 3. The computed survival time for *T*_*wat*_ considered are, respectively, 1.67, 1.96, 2.51, 6.02 and 12.17 h. This comparison with measurements indicates that the convective heat transfer coefficient between water and skin (*h*_*con*,*out*_) assumed is adequate for representing the system heat transfer.

**Fig. 4:**
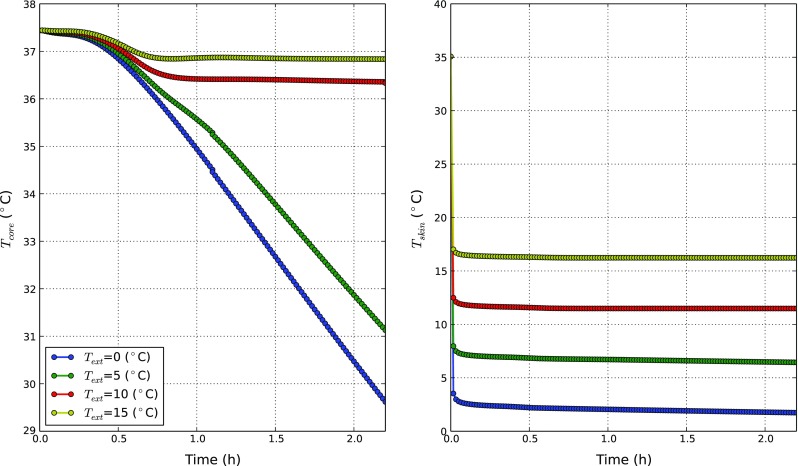
The body core and skin temperature distributions at different *T*_*wat*_. Core temperature (*left*) and skin temperature (*right*) variations

At first, we investigate the influence of water temperature on *T*_*skin*_ and *T*_*core*_. In Fig. 4, transient variations of these temperatures are presented. As seen, *T*_*skin*_ drops suddenly to very low temperatures after a few minutes. This is due to the high heat transfer coefficient, which invokes a near thermal equilibrium between the skin node and water. The rate of variation in *T*_*core*_ is much smaller but physiologically relevant. The core temperature may be considered as an indicator of the state of the inner organs. For the cases in which *T*_*wat*_ = 0.1 and 5 ^∘^C, the threshold core temperature of 35 ^∘^C is reached at around 60 and 72 min, respectively. This means that, according to Table 2, after these times, the body reaches a mild hypothermic state. We notice also that once such point is passed, core temperatures keep dropping and no steady states occur. From the physiological point of view, the body is not able to level off external losses and consequently the inner energy decreases. By contrast, for *T*_*wat*_ = 10 and 15 ^∘^C body temperature converges to steady states, and the mild hypothermia threshold is never reached. Importantly, we observe that for the initial 30 min, *T*_*core*_ exhibits very similar behaviours for different *T*_*wat*_. This response may reflect the regulatory action to temporarily prevent the drop in core temperature. All these findings are extremely important to point out the *T*_*wat*_ at which body heat balance starts to deteriorate.

**Table 2:** Hypothermia stages

	Mild	Moderate	Severe	No vital signs
*T*_*core*_ [ ^∘^C]	33–35	28–33	24–28	< 24

**Fig. 5:**
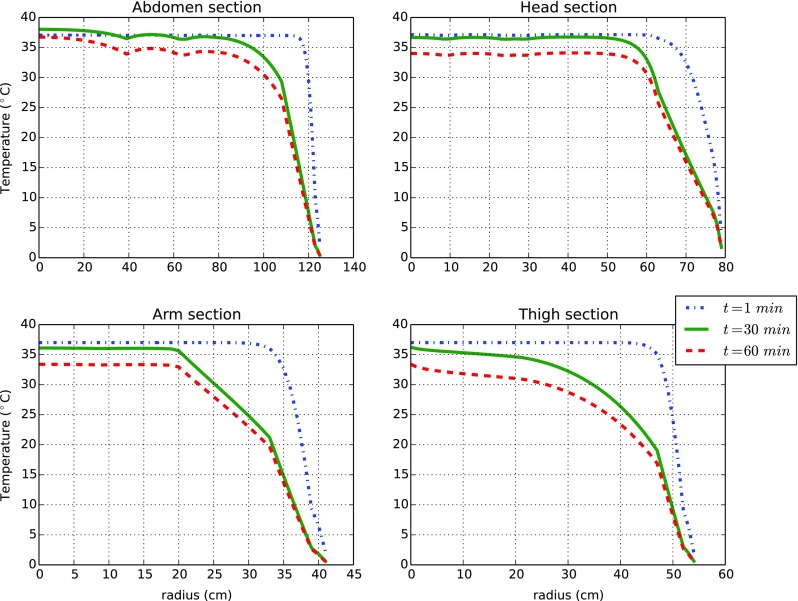
The tissue temperature distribution along the radial coordinate at various locations (sections) when exposed to water at *T*_*wat*_ = 0.1 (^∘^C). The sections considered are the ones lying in the middle (longitudinal direction) of the abdomen, head, arm and thigh cylinders

In Fig. 5, the temperature profiles in tissue at different stages of transience (1 *min*, 30 *min* and 60 *min*) for *T*_*wat*_ = 0.1 ^∘^C are presented. The tissue profiles explain clearly what is happening within the body. We note that, in all sections considered, a strong temperature gradient occurs near the skin region within the first few seconds of exposure. After such an initial cold shock, the body tries to offset the thermal losses at the skin surfaces. This behaviour is evident in the abdomen and it highlights the relevant role of the viscera metabolism in the thermal balance. Similar responses are observed for the head profiles. For the arm and thigh cylinders, the heat provided by muscle metabolism probably is insufficient, and the temperature starts to decrease more quickly than for the head and trunk ones. We note, however, that the temperature change in the abdomen is not as rapid as in other parts due to its larger cross section.

**Fig. 6:**
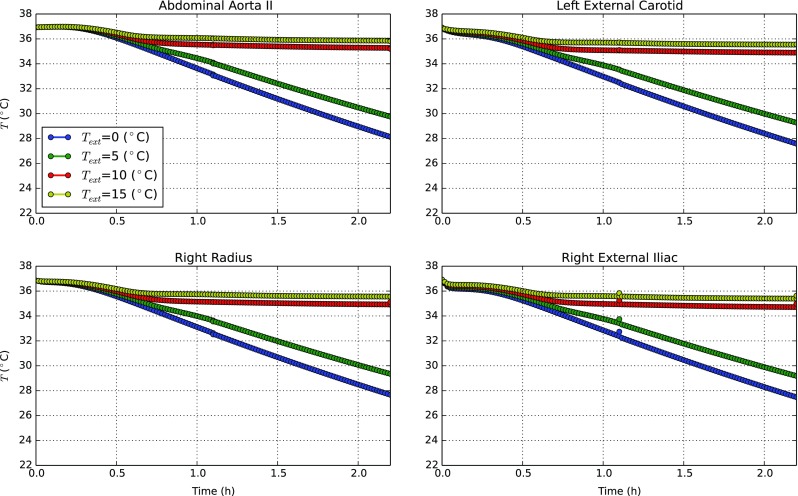
The blood temperature distribution at various blood monitoring points when exposed to water at different *T*_*wat*_

In Fig. 6, we present the blood temperature variations with time at the four selected blood monitoring points for different external conditions. As seen, the blood temperature at all monitoring points show behaviours that are inline with *T*_*core*_ variations presented in Fig. 4. These results highlight the role of the blood flow in the thermal balance. The blood indeed is not able to produce heat itself (the volumetric heat generation in the blood is irrelevant compared to the tissue thermal capacity) but it carries energy from the warmest regions to the periphery. It is possible to observe that for all blood nodes considered, there is a first stage in which temperature tends to be constant. We recollect that the blood inlet temperature depends on a thorax tissue node, and thus, as soon as trunk temperature drops, a consistent decrease in blood temperature is observed. This outcome shows how blood flow is intimately coupled with solid tissues. The temperature distributions in the ascending aorta II, left external carotid and right external iliac are almost identical. This is due to the proximity of these vessels to the thorax. Due to small size and limited resistance to heat, the temperature in the radial artery is lower than that of other arteries.

### Pathological state effects

**Fig. 7:**
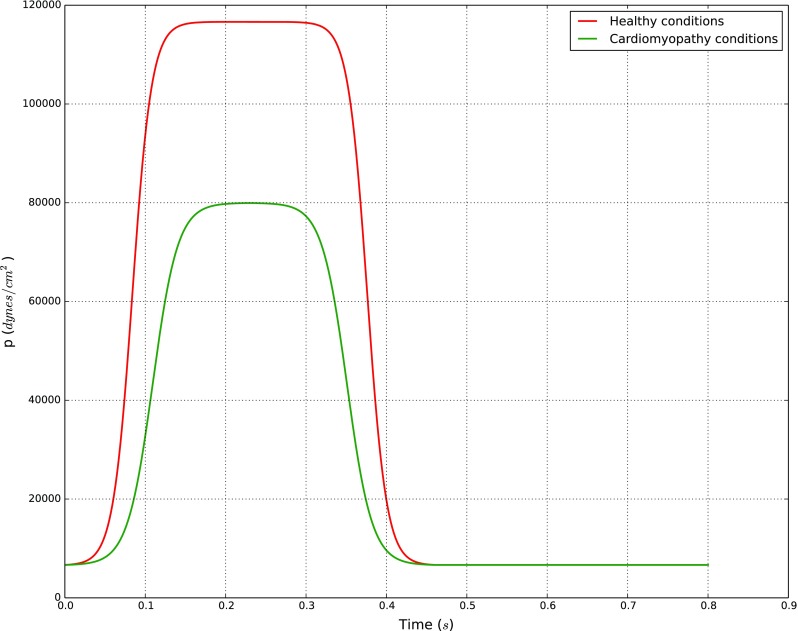
Inlet pressure at LV node for healthy and cardiomyopathy conditions. Both signals are taken from the reference work done by Mynard and Nithiarasu [10]

Here, we report the results we obtained by assuming two different disease states: cardiomyopathy and malnutrition. Cardiomyopathy is essentially a condition in which the heart’s capacity to generate force is reduced. The inlet signal at the node representing LV has been changed according to what was proposed in [10]. The peak pressure is reduced to 60 mmHg and the slope of the curves representing isovolumic contraction and relaxation phases are decreased by 30%. Figure 7 shows the comparison between inlet LV pressure wave for normal and cardiomyopathy conditions. In addition, we also assume that cardiomyopathy affects the tissue perfusion rate. It is changed proportionally with the cardiac output reduction (from 4.5 to 1.79 L/min).

Malnutrition can originate from a diet poor in vital nutrients, vitamins and minerals. Under such conditions, the total amount of energy supplied through alimentation is not sufficient to balance the physiological-energy requirements of the body. This generally leads to a weight loss because energy reservoirs stored in fat and muscles are consumed. Under such conditions, several complex biological and biochemical processes could be involved. As this goes beyond the scope of the present study, we adopted a simplified approach for modelling heat loss in malnutrition by reducing the muscle and fat tissue layer radii. The degree of weight loss was adopted from a standard clinical classification of body mass index (BMI) that ranges from a healthy individual to a subject suffering from severe anorexic symptoms [27]. For simulation purposes, a healthy body a BMI equal to 22.4 *k* *g*/*c* *m* ^2^ is assumed as control, whereas a body with BMI of 12.2 *k* *g*/*c* *m* ^2^ is considered for the case of severe malnutrition. Such a reduction is associated with 43.6% in body weight loss, consistent with clinical evidence [27]. The cylinder layer radii that correspond to these two states are reported in Table 3.

**Table 3:** Tissue layer dimensions for a body in healthy and malnutrition conditions

Cylinder	Tissues	Layer radii original (cm)	Layer radii modified (cm)
Head	Brain, bone, fat, skin	6.60, 7.60, 7.80, 8.00	6.60, 7.60, 7.80, 8.00
Neck	Bone, muscle, fat, skin	1.90, 5.40, 5.60, 5.80	1.90, 2.95, 2.97, 3.17
Thorax	Lung, bone, muscle, fat, skin	7.70, 8.90, 10.30, 12.70, 12.90	7.70, 8.90, 9.32, 9.56, 9.76
Abdomen	Viscera, bone, muscle, fat, skin	7.90, 8.30, 10.90, 12.40, 12.60	7.90, 8.30, 9.08, 9.23, 9.43
Shoulder	Bone, muscle, fat, skin	3.70, 3.90, 4.40, 4.60	3.70, 3.76, 3.81, 4.01
Arm	Bone, muscle, fat, skin	1.50, 3.40, 4.00, 4.60	1.50, 2.07, 2.13, 2.33
Forearm	Bone, muscle, fat, skin	1.50, 3.40, 4.00, 4.60	1.5, 2.07, 2.13, 2.33
Thigh	Bone, muscle, fat, skin	2.20, 4.80, 5.30, 5.50	2.20, 2.98, 3.03, 3.28
Leg	Bone, muscle, fat, skin	2.20, 4.80, 5.30, 5.50	2.20, 2.98, 3.03, 3.28

To study the effect of the disease states of cardiomyopathy and malnutrition, different external heat transfer coefficients and water temperatures are considered. They, respectively, are (0.005, 0.011, 0.05, and 0.100 *W*/(*K* *c* *m* ^2^)) and (0.1, 5, 10, and 15 ^∘^C). When water temperature is varied, *h*_*ext*_ is kept equal to 0.011 *W*/(*K* *c* *m* ^2^), while for variable *h*_*ext*_, we maintained *T*_*wat*_ = 10 ^∘^C.

**Fig. 8:**
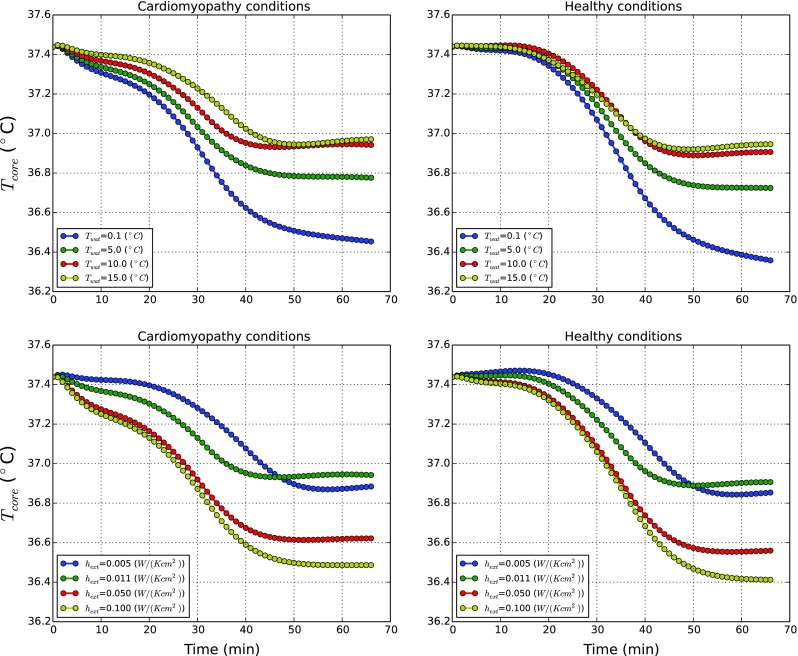
Influence of water temperature and outer surface heat transfer coefficient on a body affected by cardiomyopathy

In Fig. 8 the transition of core temperature with time for the cardiomyopathy condition are compared against healthy/standard state. For both conditions, core temperatures slightly decrease in time. The magnitude of such reductions is clearly dependent on the external water temperature and external heat transfer coefficient. The rate of reduction in the body with cardiomyopathy is lower than for the healthy body. This is due to reduced convective heat transfer as a result of slower blood flow and reduction in perfusion rate. Nevertheless the difference in temperature distributions between a human body with cardiomyopathy and normal states is small.

**Fig. 9:**
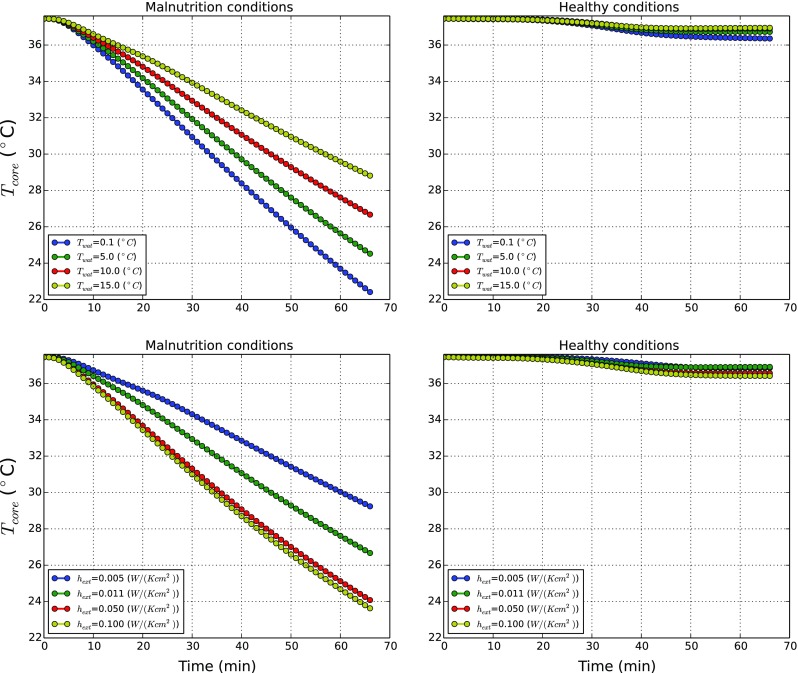
Influence of water temperature and outer surface heat transfer coefficient on a body affected by malnutrition

The results obtained for malnutrition condition on the other hand are starkly different as shown in Fig. 9. As seen for any of the *T*_*wat*_, the core temperatures decrease rapidly, reaching very low temperatures (<30 ^∘^C) within 1 h. These results show the importance of muscle and fat layers on the system thermal balance. The reduction in the muscle and fat layers has significantly impaired the system from producing sufficient heat to maintain thermal balance of the body. The effects of both disease states are investigated further using shivering and net powers generated to clearly understand the heat balance mechanism in the human body.

**Fig. 10:**
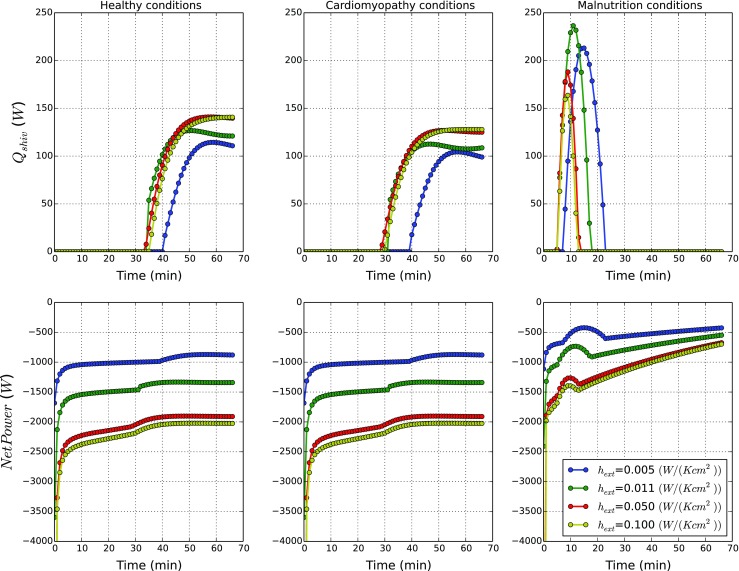
Thermal power generated by human body affected by pathological states. In the *upper part*, the global shivering, power evolutions in time are shown. In the *bottom part*, the Net Power evolutions in time are shown

Figure 10 compares the shivering (*Q*_*shiv*_) and net powers produced by normal and pathological states at different outer surface heat transfer coefficients. The Net Power is the global resultant of all power contributions (metabolism, shivering, convection, radiation, sweating and breathing) and describes how the body is thermally interacting with the environment. This quantity can be seen as the global volumetric energy production within the sytem (metabolism and shivering) minus the thermal losses at the skin (convection, radiation, sweating) and at the respiratory tract (breathing). When its value reaches zero, no more changes in body temperatures occur. A positive value of power means the metabolic generation is overwhelming the heat exchanged with environment and consequently body temperatures go up, whilst a negative value indicates that the body is cooling. For each state, we report four different *h*_*ext*_ values at *T*_*wat*_ equal to 10 ^∘^C as shown in Fig. 10.

As observed in Fig. 10, the difference in powers generated by normal and cardiomyopathy states are small. Thus, once again, we can confirm that the effect cardiomyopathy has on thermal equilibrium is negligible. In both normal and cardiomyopathy states, the shivering power generated starts when the threshold core temperatures are reached and this power is maintained at a particular rate for a long time. The net power generated for these two conditions reach different quasi-asymptotic states at different heat transfer coefficients and the heat power exchanged with the environment is almost constant. We expect such values to converge to zero after a time longer than 60 min.

The power generation under malnutrition conditions is significantly different. The shivering heat generation increases exponentially due to dramatic decrease in body temperature. The shivering heat generation then is rapidly reduced to zero as the body reached a critical state at which shivering stops. The net heat generated by the body with malnutrition appears to be higher than that of the normal or cardiomyopathy conditions. This appears to be counterintuitive but physiological. The initial net heat generation is very low, but as the core and surface temperatures are reduced, the total heat exchange with the atmosphere is also reduced (heat exchange is proportional to temperature difference). Although, as a result of this mechanism, the net power generation increases, this in no way indicating that the body is coping.

## Conclusions

The bioheat transfer model employed shows significant robustness and versatility for simulating hypothermia conditions both in healthy and pathological states. This might open up a wide array of opportunities to study problems of practical importance where clinical/experimental studies may be difficult or not possible. The model used provides a test bed for studying the impact of cardiovascular diseases and their influence on thermal balance of the human body. Many more extensions of the model are possible, including using subject-specific information to obtain more precise results related to individual patient requirements. This is particularly relevant for the recent emergence of therapeutic hypothermia or targeted temperature management (TTM) in the clinical environment for the support of cardiac arrest resuscitation and the treatment of neonatal encephalopathy. The main obstacle in employing computational techniques in this context is currently the lack of detailed experimental probing into human body thermoregulation under hypothermic conditions. The present work provides a reference framework that can be extended and elaborated in the light of further experimental evidence to provide insights into a wider spectrum of environmental conditions and potential clinical interventions. In spite of the current experimental limitations, the computational model developed was able to delineate a number of fundamental mechanisms that conform to clinical evidence. The results clearly showed that the core temperature evolution may be used as an indicator of hypothermia, which is difficult to experimentally observe. Furthermore, the temperature distribution in tissue highlights the relevant role of viscera on the thermal balance. It was also noticed that arterial blood flow may play a significant role in transporting energy to control body temperature. However, its function is overwhelmed once core temperature reaches the hypothermic threshold. On the relationship between hypothermia and pathological conditions, we have shown that cardiomyopathy conditions may not affect the thermal equilibrium of the body in any significant manner. By contrast, malnutrition subjects the body to sever thermal stress and a faster, stronger and more sustained thermoregulatory action.

## Electronic supplementary material

Below is the link to the electronic supplementary material.

(PDF 126 KB)
