# Supplementary material for: Modelling accidental hypothermia effects on a human body under different pathophysiological conditions
Source: Med Biol Eng Comput. 2017 Jun 5;55(12):2155–67. doi: 10.1007/s11517-017-1657-3 (PMC5680406; doi:10.1007/s11517-017-1657-3)
Supplement: Supplementary file 1 — (PDF 126 KB) [file 11517_2017_1657_MOESM1_ESM.pdf]

tex file Click here to download Manuscript hypo_appendix.tex
Click here to view linked References

Supplementary material

Respiration and thermoregulatory equations

To estimate breathing losses, the following formulation has been used [1].

$$q_{bre} = \frac{1}{V_{lung}}[0.0014(m^2/^oC)Q_{m,glob}(34 - T_{out}) + 0.0173(m^2/Pa)Q_{m,glob}(5.87 - P_{out})] \quad (1)$$

where $Q_{m,glob}$ is the global metabolic heat generation rate, V_{lung} is the lung volume (respectively 58.2 W/m^2 and 5631.41 cm^3) and P_{out} is the ambient water vapour pressure.

Both cutunaeous vasomotion and shivering are modeled following [1]. The evaluation of vasodilation and vasoconstriction flows ($\dot{m}_{skin,dil}$ and $\dot{m}_{skin,con}$) for a body segment may be calculated via the following expressions:

$$\begin{cases} \dot{m}_{skin,dil} = \dot{m}_{skin,bas}\ kg/s \quad if \quad T_{core} \leq 36.8^oC \\ \dot{m}_{skin,dil} = \frac{T_{core}-36.8}{37.2-36.8}(\dot{m}_{skin,max} - \dot{m}_{skin,bas}) + \dot{m}_{skin,bas}\ (kg/s) \quad if \quad 36.8^oC \leq T_{core} \leq 37.2^oC \\ \dot{m}_{skin,dil} = \dot{m}_{skin,max}\ kg/s \quad if \quad T_{core} \geq 37.2^oC \end{cases} \quad (2)$$

and

$$\begin{cases} \dot{m}_{skin,con} = \dot{m}_{skin,min}\ kg/s \quad if \quad T_{skin} \leq 27.8^oC \\ \dot{m}_{skin,con} = \frac{T_{skin}-27.8}{33.7-27.8}(\dot{m}_{skin,bas} - \dot{m}_{skin,min}) + \dot{m}_{skin,min}\ (kg/s) \quad if \quad 27.8^oC \leq T_{skin} \leq 33.7^oC \\ \dot{m}_{skin,con} = \dot{m}_{skin,bas}\ kg/s \quad if \quad T_{skin} \geq 33.7^oC \end{cases} \quad (3)$$

For evaluating shivering contribution, we need at first calcuate the shivering temperature, T_{shiv}; this variable depends on the core temperature:

$$\begin{cases} T_{shiv} = 35.5^oC \quad if \quad T_{core} \leq 35.8^oC \\ T_{shiv} = -10222 + 570.9\ T_{core} - 7.9455\ T_{core}^2\ (^oC) \\ \quad if \quad 35.8^oC \leq T_{core} \leq 37.1\ ^oC \end{cases} \quad (4)$$

It should be noted that for T_{core} greater than 37.1^oC, shivering does not occur. The maximum increase in total metabolic heat generation caused by shivering ($Q_{shiv,max}$) may be written as

$$Q_{shiv,max} = \frac{1}{3600}(-1.186110^9 + 6.55210^7\ T_{core} - 9.041810^5\ T_{core}^2)\ (W) \quad (5)$$

The shivering metabolic heat generation Q_{shiv} may now be calculated as

$$Q_{shiv} = Q_{shiv,max}[1 - (\frac{T_{skin} - 20}{T_{shiv} - 20})^2] \ (W) \quad if \quad (40 - T_{shiv}) \leq T_{skin} \leq T_{shiv} \ ^oC \quad (6)$$

In the following we reported the methodology used for describing sweating losses. The sweating threshold T_{eva} may be approximately expressed as a function of mean skin temperature, i.e.,

$$\begin{cases} T_{eva} = 42.084 - 0.15833 \ T_{skin} \ (^oC) \\ \ \ if \ \ T_{skin} \leq 33.0^oC \\ T_{eva} = 36.85^oC \ \ if \ \ T_{skin} \geq 33.0^oC \end{cases} \quad (7)$$

The sweat rate $\dot{m}_{eva}$ may now be evaluated as,

$$\dot{m}_{eva} = \frac{45.8 + 739.4(T_{core} - T_{eva})}{3.6 \ 10^6} \quad (kg/s) \quad if \quad T_{core} > T_{eva} \quad (8)$$

The relative skin wetness w is given as,

$$w = 0.06 + \frac{\dot{m}_{eva}(1 - 0.06)}{0.000193} \quad (9)$$

The total evaporative heat loss q_{eva} may now be written as [2],

$$q_{eva} = \frac{w(P_{skin} - P_{out})}{R_{eva,cl} + \frac{1}{f_{cl} h_{eva}}} \ (W/m^2) \quad (10)$$

where P_{skin} is water vapour pressure on the skin (normally assumed to be that of saturated water vapour at skin temperature [3]), $R_{eva,cl}$ is the evaporative heat transfer resistance of the clothing layer, f_{cl} is the clothing area factor (the surface of the clothed body divided by the area of the bare body), and h_{eva} is the evaporative heat transfer coefficient (see [4]).

References

[1] C. Smith. *A transient, Three Dimensional Model of the Human Thermal System.* PhD thesis, Kansas State University, 1991.

[2] Refrigerating American Society of Heating and Air-Conditioning Engineers. Physiological principles and thermal comfort, 1993.

[3] P.O. Fanger. *Thermal Comfort.* New York: McGraw-Hill, 1970.

[4] D.M. Kerslake. *The Stress of Hot Environments.* Cambridge: University Press, 1972.
